# The Impact of Health‐Promoting Lifestyle Behaviors on Gut Microbiota in Survivors of Hematological Cancer: A Scoping Review

**DOI:** 10.1002/cnr2.70224

**Published:** 2025-05-13

**Authors:** Elham Samami, Angela Starkweather, Debra Lynch Kelly, Debra Lyon

**Affiliations:** ^1^ College of Nursing University of Florida, Health Cancer Center Gainesville Florida USA; ^2^ College of Nursing Rutgers University Newark New Jersey USA; ^3^ Loewenberg College of Nursing University of Memphis Memphis Tennessee USA

**Keywords:** gut microbiota, health‐promoting lifestyle behaviors, hematological cancer, nutrition, survivorship

## Abstract

**Purpose:**

This scoping review aims to explore the relationship between health‐promoting lifestyle behaviors and gut microbiota in survivors of hematological cancers, including leukemia, lymphoma, and multiple myeloma. Given the rising incidence of these malignancies and the associated treatment challenges, understanding how lifestyle factors influence gut health may provide insights into improving survivorship outcomes.

**Methods:**

We conducted a comprehensive search across multiple databases, including PubMed/Medline, CINAHL, and Scopus, following the Preferred Reporting Items for Systematic Reviews and Meta‐Analysis extension for Scoping Reviews (PRISMA‐ScR). The search strategy incorporated MeSH terms related to hematological cancers, health‐promoting lifestyle behaviors, and gut microbiota. Inclusion criteria focused on primary research studies published in English that reported gut microbiota results in survivors of hematological cancers. A total of 1717 papers were initially identified, with 16 studies meeting the inclusion criteria after screening for relevance.

**Results:**

The review identified a significant association between health‐promoting lifestyle behaviors, such as physical activity, nutrition, and stress management, and the composition and diversity of gut microbiota in cancer survivors. The findings suggest that engaging in these behaviors may enhance gut health, potentially mitigating treatment‐related symptoms and improving overall quality of life. Notably, the studies highlighted the importance of tailored interventions that consider individual patient needs and preferences.

**Conclusions:**

This scoping review underscores the critical role of health‐promoting lifestyle behaviors in influencing gut microbiota among survivors of hematological cancers. Future research should focus on longitudinal studies to establish causal relationships and explore the mechanisms underlying these associations. By promoting healthy lifestyle choices, healthcare providers can enhance survivorship care and improve health outcomes for this population.

## 1 Introduction

Hematological malignancies encompass a diverse array of cancer types, including leukemia, lymphoma, and multiple myeloma. Since 1990, the incidence of these cancers has been increasing, with an estimated 141 360 new cases and 43 810 deaths in 2024 [1]. Conversely, the mortality rates for all forms of hematologic malignancies have been consistently declining, increasing the number of long‐term survivors. The medical treatments that have led to these improvements in outcomes, however, also present numerous challenges for survivors across physical, psychological, and social domains [2]. Managing these treatments' symptoms and side effects require meticulous care and assessment. One area of growing interest is the role the gut microbiota plays in modulating these symptoms [3]. A complex community of microorganisms residing in the gastrointestinal tract, the gut microbiota plays a crucial role in maintaining overall health. It is involved in a variety of functions including digestion, immune regulation, and protection against pathogens. A healthy gut microbiota contributes to the efficient absorption of nutrients, production of essential vitamins and enzymes, maintenance of a robust immune system and gut barrier function, and metabolism of dietary compounds. It also influences mood, behavior, and cognitive function via the gut‐brain axis [4, 5, 6]. Hematological cancer treatments like chemotherapy, stem cell transplantation, and antibiotics can disrupt the gut microbiota, leading to dysbiosis and loss of diversity [3, 7, 8]. Dysbiosis refers to changes or imbalances in microbial communities' structure, composition, and function. This definition acknowledges that, because of significant variation between individuals, there is no universal standard for identifying the ideal composition of a healthy gut microbiota [9].

These changes in the gut microbiota harm gastrointestinal (GI) symptoms and psychosocial health and are associated with poor outcomes like fatigue and depressive symptoms during cancer treatment and, eventually, increased risks of mortality and stress‐induced gut microbiota dysbiosis by graft‐versus‐host disease (GvHD) [3, 8, 10].

The gut microbiota thus represents a promising area for the development of strategies to enhance symptom management and improve overall quality of life for survivors of hematological malignancies. Health‐promoting lifestyle behaviors are relatively straightforward targets for intervention, and the delivery of effective and reliable advice relies not only on the available research but also on the current understanding of various health‐promoting lifestyle behaviors [11, 12]. Diet is one of the most significant factors influencing gut microbiota composition. A balanced diet rich in fiber, fruits, vegetables, and fermented foods promotes diversity and an abundance of beneficial species in the gut microbiota. Conversely, diets high in processed foods and saturated and monounsaturated fats can lead to dysbiosis and an inflammatory state in the gut [13, 14]. In addition to a balanced diet, regular exercise positively influences gut microbiota composition, enhancing the abundance of beneficial bacterial species involved in amino acid biosynthesis, carbohydrate metabolism, and short‐chain fatty acid (SCFA) production [15, 16]. Effective stress management is another critical component of maintaining gut health. Chronic stress can negatively impact the gut microbiota via the gut‐brain axis, resulting in increased gut inflammation and permeability and allowing bacteria and toxins to leak into the bloodstream, further exacerbating systemic inflammation [17].

Health responsibility, spiritual growth, and interpersonal relationships also have significant effects on gut microbiota composition and diversity [17, 18, 19]. Health responsibility, or an active sense of accountability for one's own well‐being, includes paying attention to one's own health, educating oneself about health, and exercising informed consumerism when seeking professional assistance [18]. Health responsibility plays a pivotal part in shaping gut microbiota, primarily through dietary choices, exercise, and the maintenance of mental well‐being [20].

Spiritual growth, involving practices like long‐term meditation, has been shown to increase the abundance of beneficial bacterial genera such as *Megamonas* and *Faecalibacterium*, which are linked to reduced risks of mental and physical ailments [19, 21]. Strong interpersonal relationships, especially in the context of marriage, contribute to the richness (i.e., number of species) of the microbial community [22]. One study reported that married individuals, particularly those in high‐quality, close relationships, possessed a more varied gut microbiota than individuals who lived alone, underscoring the role of close interpersonal relationships in promoting gut health [22]. These studies collectively highlight the links between lifestyle, emotional well‐being, and gut microbiota, suggesting that a holistic approach to health can profoundly influence gut health and overall well‐being.

Targeted interventions that foster or increase integrative health‐promoting lifestyle behaviors can mitigate the negative effects of cancer treatments on gut health and support the long‐term well‐being of cancer survivors [23].

Despite advancements in treatment leading to improved survival rates, the long‐term health of these survivors is often compromised by disruptions to gut microbiota due to chemotherapy, stem cell transplantation, and antibiotic use [24]. Research, however, has tended to focus on individual lifestyle factors in isolation without considering their combined effects on gut health and overall well‐being. By synthesizing existing evidence and highlighting the interconnectedness of these health‐promoting lifestyle behaviors, we aim in this scoping review to provide valuable insights regarding the holistic impact of these behaviors on the gut microbiota and the role of the gut microbiota in modulating symptoms and side effects associated with treatments for hematological malignancies. Our goal is to support the development of targeted strategies to enhance symptom management and improve long‐term well‐being for survivors of these cancers.

## 2 Conceptual Framework

**FIGURE 1:**
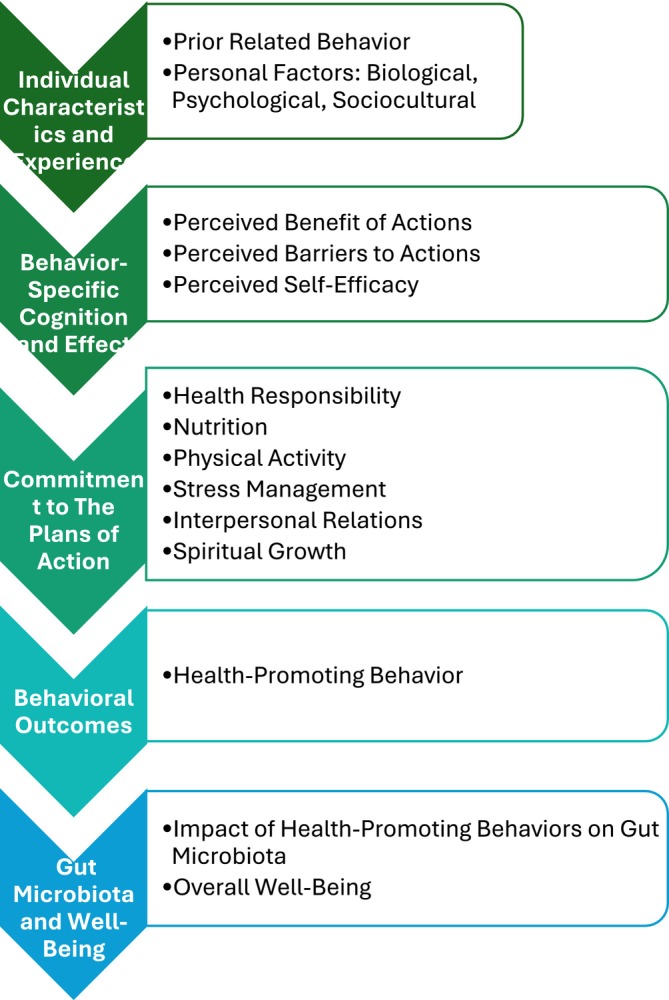
Gut microbiota well‐being through health‐promoting behavior from Pender's Health Promotion Model (HPM) and the Health‐Promoting Lifestyle Profile II (HPLPII). The HPM focuses on the complex interplay between individual characteristics and experiences, behavior‐specific cognitions and effects, health‐promoting behaviors, and well‐being.

We adapted Pender's health promotion model (HPM) to serve as the framework for this review (Figure 1) [25]. This model proposes an integrative approach to achieving a healthy microbiome and well‐being by illustrating how previous experience and personal biological, psychological, and sociocultural factors shape behavior‐specific cognition, including perceptions of barriers and facilitators, which can lead to an internal commitment to behavior and result in behavioral changes that improve well‐being. We have adapted the HPM to focus on domains of health‐promoting behaviors measured by the Health‐Promoting Lifestyle Profile II (HPLP II), including nutritional habits, social connections, exercise, stress management, health responsibility, and spiritual well‐being. The HPLP II is a strong predictor of control over health, personal competence, and health status. We used this adapted model as a tool for mapping research findings reported in the literature to assess whether the current body of research adequately addressed each domain of health‐promoting behavior as the antecedents of healthy gut microbiota in hematological cancers and to identify critical gaps that must be addressed to develop an effective approach to improving gut microbiota health and related health outcomes in hematological cancer survivors.

## 3 Methods

We followed the Preferred Reporting Items for Systematic Reviews and Meta‐Analysis extension for Scoping Reviews (PRISMA‐ScR) in our conduct of this review [26], starting with a comprehensive search of PubMed/Medline, the Cumulative Index to Nursing and Allied Health Literature (CINAHL), and Scopus. In consultation with a research librarian, we developed a search strategy using MeSH terms that encapsulated hematological cancers (leukemia, lymphoma, and multiple myeloma); all dimensions of the HPLP II (health self‐responsibility, nutrition, physical activity, stress management, interpersonal relationships, spiritual growth); and the gut microbiota. The final search strategy for all of the databases can be found in Table S1.

The inclusion criteria for this scoping review required studies that met the following conditions: (1) they focused on survivors of hematological cancer, (2) they were published in English, and (3) they reported gut microbiota results for the participants. Additionally, (4) only studies with a quantitative design were included, such as cross‐sectional, observational, quasi‐experimental, cohort, or randomized controlled trials (RCTs). There was no restriction on the specific measures or methods used to assess gut microbiota health. (5) Published dissertations and conference posters were also considered for inclusion. In contrast, the exclusion criteria eliminated studies that met any of the following conditions: (1) nonclinical and in vitro studies, (2) study protocols, (3) systematic reviews, (4) editorials, or (5) letters to the editor. The review did not include studies that were not part of the database search, but (6) the authors supplemented their search by exploring the gray literature and scanning the reference lists of the included studies. The final search was conducted at the end of December 2024.

The selection process for the included articles involved three screening stages: title, abstract, and full text. One author (ES) conducted the title, abstract, and full‐text screening. The following data were extracted from the papers selected for inclusion in this review: author names; publication date; study aim, design, and population; sample size; and any associations identified between health‐promoting lifestyle behaviors and gut microbiota.

## 4 Results

**FIGURE 2:**
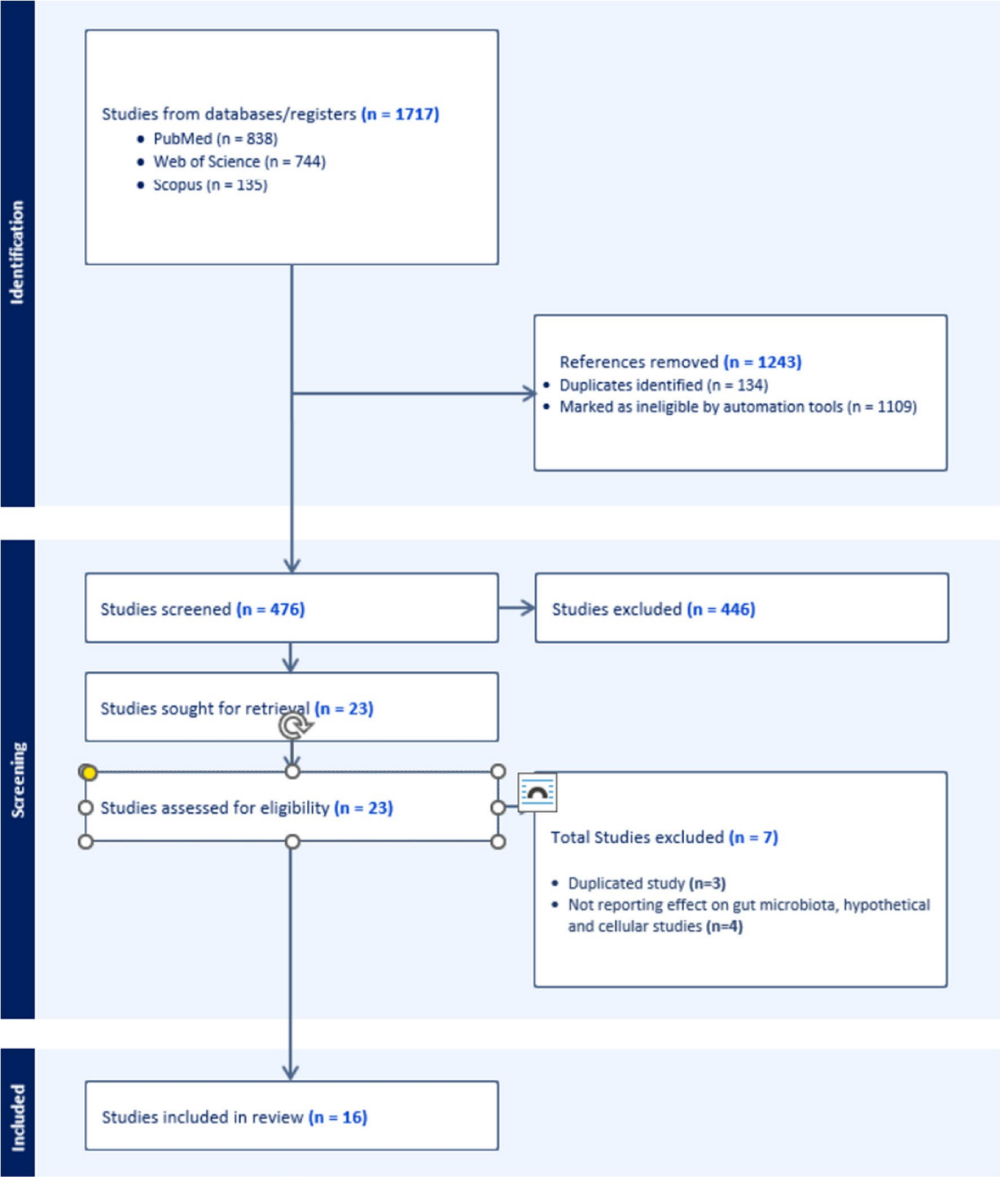
PRISMA diagram of the search procedure.

Our initial search returned 1717 papers. After we removed duplicates and screened titles, abstracts, and full texts for compliance with our inclusion and exclusion criteria, 16 papers remained for review (see Figure 2). All papers included assessed the association between health‐promoting lifestyle behaviors and gut microbiota alterations in hematological cancer survivors (Table S1) (Table 1). Most of the studies were performed in the United States (*n* = 6), with others conducted in Japan (*n* = 2), Italy (*n* = 1), Slovakia (*n* = 1), Mexico (*n* = 1), Norway (*n* = 1), Australia (*n* = 1) and one multi‐site study. Each study focused on a single domain of the HPLP II, with 13 examining nutrition and one examining physical activity. None assessed health self‐responsibility, stress management, interpersonal relationships, or spiritual growth in relation to the gut microbiota of hematological cancer survivors. Study designs varied and included retrospective comparative studies, longitudinal studies, RCTs, pilot studies, prospective cohort studies, and observational studies. Sequencing of the 16S rRNA gene was the only method used to examine microbiota in 13 studies, with the remaining study reporting a change in gut microbiota translocation. PEVuZE5vdGU [27].

**TABLE 1:** Summary of study characteristics (*N* = 15).

Author, year	Country	Aim	Design	Sample	Population	HPLP II domain	Stat method	Effect on gut microbiota	Health outcome
Iyama et al., 2014 [27]	Japan	To investigate whether EN with GFO before and after HSCT ameliorates gut injury induced by HSCT conditioning regimens and improves short‐term survival.	Retrospective comparative study with a matched‐pair control group	*N* = 44 patients who underwent HSCT at a single hospital between January 2009 and April 2011; *n* = 22 received EN, *n* = 22 controls did not	Median age of 52.5	Nutrition	Linear discriminant analysis effect size, Kruskal‐Wallis Test, ANOVA	GFO reduced translocation of *Enterococcus* species compared to the control group, but difference was not statistically significant (*p* = 0.07).	EN with GFO before and after HSCT reduced severity and duration of diarrhea and oral mucositis compared to no‐EN controls. EN group had better weight loss and fewer days of IV nutrition and better survival at Day 100 (100% vs. 77.3%; *p* = 0.009) than controls. The authors proposed that EN with GFO reduced gut microbial dysbiosis.
D'Amico et al., 2019 [28]	Italy	To evaluate the effect of EN vs. PN on the recovery of gut microbiota composition and function in pediatric patients undergoing HSCT.	Longitudinal study analyzing fecal samples	*N* = 20 pediatric patients undergoing HSCT; *n* = 10 received EN, *n* = 10 received PN	EN (mean age 9.3 years) PN (mean age 10.1 years)	Nutrition	Wilcoxon rank‐sum test, unifrac Permutation tests (Adonis) Kruskal‐Wallis Test	EN promoted prompt recovery of a diverse, eubiotic gut microbiota layout after HSCT. EN group had higher abundance of SCFA‐producing bacteria like *Lachnospiraceae, Blautia, Dorea, Parabacteroides*, and *Oscillospira* after HSCT and higher levels of SCFAs like butyrate, acetate and propionate in fecal samples.	EN may reduce risk of systemic infections and GvHD by supporting a eubiotic microbiota and SCFA production. No EN patients had bloodstream infections, while 7 PN patients developed 11 bloodstream infections. Prompt microbiota recovery in EN patients may have helped boost immunological reconstruction and protect against GvHD.
Ugrayová et al., 2022 [29]	Slovakia	To identify the effects of HSCT on gut microbiota composition and examine the association between intra‐hospital physical exercises after HSCT and gut microbiota structure in children with ALL.	Prospective cohort study	*N* = 29; *n* = 16 pediatric patients with ALL who underwent HSCT, *n* = 13 healthy controls	Aged 7–19 years	Physical activity	Spearman's Rank Correlation Coefficient, One‐way ANOVA, Principal Component Analysis (PCA)	Negative effect of HSCT on bacterial diversity and richness, with an increase in relative abundance of pathogenic bacteria like *Enterococcus* spp., and *Klebsiella* spp. *Enterococcus* spp. abundance positively associated with C‐reactive protein levels. Bacterial diversity is positively associated with exercise training number of sessions and total volume of exercise.	Changes in microbiota after HSCT associated with systemic inflammation.
Stein‐Thoeringer et al., 2019 [30]	United States, Germany, Japan	To investigate the role of the gut bacterium *Enterococcus* in GvHD after allo‐HCT.	Multicenter cohort study with allo‐HCT patients combined with experimental mouse models.	*N* = 1325 adult allo‐HCT recipients across 4 centers	Mean 54.48 years	Nutrition	ANOVA/Kruskal‐Wallis Test, LEfSe, Bray–Curtis Dissimilarity and UniFrac Distances, Cox Proportional Hazards Model	*Enterococcus* growth dependent on lactose as a nutrient source. Depletion of dietary lactose attenuated *Enterococcus* outgrowth in mice.	*Enterococcus* domination of gut microbiota increased risk of acute GvHD and overall/GvHD‐related mortality in allo‐HCT patients. Lactose‐free diet reduced *Enterococcus* expansion and mitigated GvHD in mouse models. Patients with lactose non‐absorber genotypes showed prolonged *Enterococcus* domination after antibiotics.
Reyna‐Figueroa et al., 2019 [31]	Mexico	To assess the effects of probiotic supplementation with *Lactobacillus rhamnosus* GG on chemotherapy‐induced gastrointestinal side effects in children with acute leukemia (AL).	Randomized, single‐blind, pilot study	*N* = 60 children (< 17 years of age) diagnosed with AL who were on remission induction or remission reinduction chemotherapy; *n* = 30 received probiotics; *n* = 30 no‐probiotics controls	Patients under 17 years of age years	Nutrition	Student test χ2 test	N/A	Probiotic supplementation decreased prevalence and severity of chemotherapy‐induced gastrointestinal side effects like nausea, vomiting, abdominal distension, diarrhea, and constipation compared to the control group.
D'Angelo et al., 2023 [32]	USA	To evaluate the associations between dietary fiber intake and diet quality, gut microbiota composition, and outcomes after autologous stem cell transplantation in patients with multiple myeloma.	Prospective study	*N* = 30 patients with multiple myeloma undergoing autologous stem cell transplantation	Median 64 years	Nutrition	LefSe, Bray–Curtis Dissimilarity, Bray–Curtis Dissimilarity, Wilcoxon Rank Sum Test:	Patients in the highest quartile of dietary fiber intake had the highest alpha diversity of the gut microbiota diversity pre‐transplant, but it was not significantly higher than those in the lowest quartile. Broad‐spectrum antibiotic use in 24/30 (80%) patients likely impacted gut microbiota community structure early after transplant.	Lower gut microbiota diversity at engraftment was associated with inferior treatment response after autologous stem cell transplantation (partial response vs. very good partial response/complete response).
Shah et al., 2022 [33]	USA	To evaluate the relationship between dietary factors, stool metabolites, and microbial composition and sustained MRD negativity in multiple myeloma patients on lenalidomide maintenance therapy.	Prospective study	*N* = 74 patients with multiple myeloma eligible for maintenance therapy during first‐line treatment; various subsets with dietary, microbiome, and metabolite data available	> 65 years	Nutrition	Wilcoxon Rank Sum Test, Spearman's Rank Correlation Coefficient Multivariable Logistic Regression	Dietary factors like higher intake of seafood, plant protein, and flavonoids and greater diversity of flavonoid intake correlated with higher stool butyrate levels and greater microbiome diversity.	Higher stool butyrate levels, abundance of butyrate‐producing bacteria, and greater microbiome alpha diversity at 3 months were associated with sustained MRD negativity in multiple myeloma patients on maintenance therapy.
Shah et al., 2024 [34]	USA	To investigate the impact of a HFPBD on biomarkers of disease and progression from MGUS/SMM to MM.	Pilot Single‐arm trial	*N = 20* Patients with MGUS/SMM	N/A	Nutrition	Wilcoxon Rank Sum Test, Spearman's Rank Correlation Coefficient, Multivariable Logistic Regression	Increased alpha‐diversity and butyrate producers	Improved BMI, insulin resistance, adiponectin leptin ratio, decreased inflammation and increased anti‐inflammatory classical monocyte
Ladas et al., 2016 [35]	USA	To evaluate the safety and feasibility of administering the probiotic LBP to children and adolescents undergoing allogeneic HCT.	Pilot study	*N* = 30 evaluable children and adolescents (aged 2–17 years) undergoing first myeloablative allogeneic HCT	Aged 2–17 years	Nutrition	Bray–Curtis Dissimilarity and UniFrac Distance, PERMANOVA (Permutational Multivariate Analysis of Variance)	The study did not directly measure the effect on the overall gut microbiota composition. However, they screened a subset of 22 patients for colonization of LBP in the stool and found that 21 had at least one stool specimen positive for LBP, suggesting gut colonization with LBP administration.	Primary safety endpoint: 0% incidence of LBP bacteremia. Secondary endpoints: feasibility of administration (97% received ≥ 50% of probiotic dose); non‐LBP bacteremia (20%), new‐onset *Clostridium difficile* infection (20%); and acute GvHD incidence (30% by Day 100, with 17% Grade 2 and 13% Grade 3).
Paredes et al., 2024 [36]	USA	To investigate the effects of dietary fiber on aGVHD after allo‐HCT	Pre‐clinical mouse model and longitudinal human study	*N = 173 allo‐HCT patients for dietary data; N* = 180 mice for preclinical model.		Nutrition	N/A	Higher microbial α‐diversity, higher abundance of butyrate producers, and increased concentrations of short‐chain fatty acids (SCFAs).	Better overall survival and lower cumulative incidence of lower GI‐aGVHD in allo‐HCT patients.
Yoshifuji, et al., 2020 [37]	Japan	To evaluate the ability of prebiotics to mitigate complications after allo‐HCT, including acute GvHD, by alleviating mucosal damage and manipulating the gut microbiota.	Prospective study	*N* = 191 patients undergoing, or having undergone, allo‐HCT; *n* = 49 receiving prebiotics; *n* = 142 historical control group	Mean 50.8 years	Nutrition	Mann–Whitney U Test, Fisher's Exact Test, Gray's Test	Prebiotic intake preserved microbial diversity and the population of butyrate‐producing bacteria in the gut after allo‐HCT compared to the control group. Fecal butyrate concentration was maintained or increased more frequently in the prebiotics group.	Prebiotic intake mitigated oral mucositis and reduced the duration of diarrhea in the early phase after allo‐HCT. The incidence of all grades of acute GvHD and Grades 2–4 acute GvHD was significantly lower in the prebiotics group compared to the control group. The cumulative incidence of acute skin GvHD was markedly decreased in the prebiotic group compared to controls.
Gorshein et al., 2017 [38]	USA	To investigate whether supplementation with the probiotic *Lactobacillus rhamnosus* GG can modify the gut microbiome and reduce the incidence of GvHD after allo‐HCT.	Randomized clinical trial	*N* = 31 patients undergoing allo‐HCT; *n* = 20 received probiotics; *n* = 11 did not (control group)	Mean 52.8 years	Nutrition	Wilcoxon Rank‐Sum Test, DeSeq2, Zero‐Inflated Beta Random Effect Model (ZIBR)	Probiotic supplementation did not appreciably alter gut microbiome diversity or composition compared to controls. Specifically, there was no increase in *Lactobacillus* abundance in the probiotic group.	No significant difference in the incidence or severity of GvHD between the probiotic and control groups after allo‐HCT. However, in the control group, higher abundance of bacteria like *Blautia* and *Ruminococcus* was associated with reduced incidence of GvHD, corroborating previous findings on a protective role of certain gut bacteria against GvHD.
Andersen et al., 2020 [39]	Australia	To determine if there is a difference in the gastrointestinal microbiome between patients receiving EN vs. PN after allo‐HCT.	Pilot study as part of a randomized controlled trial investigating tolerability of early EN vs. standard care (PN when required) post‐allo‐HCT	*N* = 23 patients aged ≥ 18 years undergoing allo‐HCT for hematological conditions; *n* = 13 received predominantly EN; *n* = 10 received predominantly PN; stool samples collected on Day 30 post‐transplant	Median 52 years	Nutrition	PERMANOVA, Kruskal‐Wallis Test, Mann–Whitney U Test, Fisher's Exact Test	No difference in microbial diversity between EN and PN groups. EN group had greater abundance of beneficial taxa like *Faecalibacterium, Alistipes, Agathobacter, Roseburia, Anaerostipes*, and *Ruminococcus*. PN group had a greater abundance of *Proteobacteria, Klebsiella*, and *Enterococcus*. Longer duration of minimal oral intake associated with different overall profile, lower diversity, and lower SCFA‐producing taxa.	The study did not directly measure health outcomes, but authors suggest that the EN‐associated microbiome profile with more SCFA‐producing taxa may promote better clinical outcomes based on previous evidence linking the gut microbiome to transplant complications and survival.
Andermann et al., 2021 [40]	USA	To determine the maximum tolerated dose, feasibility of administration, and toxicity of the prebiotic FOS in patients undergoing reduced‐intensity allo‐HCT. To characterize the effects of FOS on gut microbiome composition, stool SCFA concentrations, and peripheral Treg concentrations.	Phase I pilot, single‐arm, dose‐escalation clinical trial	*N* = 31 adults (> 18 years old) with hematologic malignancies undergoing reduced‐intensity allo‐HCT; *n* = 15 received FOS starting at pre‐transplant conditioning and continuing for 21 days total; *n* = 16 control patients not receiving FOS; stool and blood samples collected from both groups	Mean 61 years	Nutrition	ADONIS Test, Student's T‐test, Shannon Diversity Index, Mixed Linear Models	FOS intake was associated with community‐level taxonomic differences in the gut microbiome on the day of transplant compared to controls. Differences did not persist beyond the day of transplant. FOS was not associated with significant changes in stool SCFA concentrations at Day 14 post‐transplant.	The maximum tolerated dose of FOS was determined to be 10 g/day, with transient effects on the gut microbiome and possible immunomodulatory effects on Tregs, limited primarily by nausea and mucositis. No significant differences were found between the FOS and control groups in rates of acute GvHD, bloodstream infections, *C. difficile* infections, or overall mortality. Patients receiving FOS had higher peripheral blood concentrations of CTLA4+ CD4+ activated T cells (likely Tregs) at Day 28 post‐transplant compared to controls.
Riwes et al., 2023 [41]	USA	To determine the clinical feasibility of administering RPS as a prebiotic dietary intervention and its effect on the intestinal microbiota and its dependent metabolites in allo‐HCT recipients.	Prospective interventional study	*N* = 10 adults undergoing human leukocyte antigen‐matched, related‐donor myeloablative allo‐HCT	Median 57 years	Nutrition	ANCOM (Analysis of Composition of Microbiomes), LEfSe, Mixed Random Effects Model, DADA2	RPS supplementation significantly increased intestinal butyrate levels, a byproduct of the microbial metabolism of RPS. The intervention altered the intestinal microbial metabolite butyrate.	The study provides preliminary evidence that RPS supplementation is a feasible way to favorably modulate the gut microbiome and metabolites in allo‐HCT recipients, despite the use of HCT‐related medications like antibiotics. No adverse effects attributed to RPS were observed.
Skaarud et al., 2021 [42]	Norway	To investigate whether a nutritional intervention by providing optimized energy and protein intake influenced gut microbiota, SCFAs, and markers of gut barrier functions in allo‐HCT patients, and if these parameters were associated with clinical outcomes.	Secondary analysis of a randomized controlled trial	*N* = 47 adults (≥ 18 years old) undergoing allo‐HCT after myeloablative conditioning for a hematological malignancy; *n* = 23 who received the nutritional intervention; *n* = 24 who did not	Median 45 years	Nutrition	PERMANOVA, Wilcoxon Signed Rank Test, Receiver Operating Characteristic (ROC) Curves, Kaplan–Meier Plots, Spearman's Test	The nutritional intervention had no significant effect on gut microbial diversity, SCFA concentrations, or markers of gut barrier function as compared to the control condition. Significant reductions were seen in both groups from baseline to 3 weeks post‐transplant in microbial diversity, concentrations of most SCFAs, and some markers of gut barrier function.	1‐year mortality was significantly higher in patients with lower microbial diversity and *Blautia* abundance at 3 weeks post‐transplant. Fecal propionic acid levels were associated with 1‐year survival. Markers of gut barrier function were less strongly associated with clinical outcomes. No significant associations were found with acute GvHD. The nutritional intervention did not significantly mitigate the negative changes in gut microbiota and clinical outcomes after allo‐HCT.

Abbreviations: AL = acute leukemia; ALL = acute lymphoblastic leukemia; allo‐HCT = allogeneic hematopoietic stem cell transplantation; EN = enteral nutrition; FOS = fructooligosaccharides; GFO = glutamine, fiber, and oligosaccharides; GvHD = graft‐versus‐host disease; HCT = hematopoietic cell transplantation; HFPBD = high fiber plant‐based dietary; HSCT = hematopoietic stem cell transplantation; ICB = immune checkpoint blockade; LBP = *Lactobacillus plantarum* ; LEfSe = linear discriminant analysis effect size; MGUS = monoclonal gammopathy of undetermined significance; MM = multiple myeloma; MRD = minimal residual disease; PERMANOVA = Permutational Multivariate Analysis of Variance; PN = parental nutrition; RPS = resistant potato starch; SCFA = short‐chain fatty acid; SMM = smoldering multiple myeloma; Treg = regulatory T cell.

The selected studies employed a variety of participant sampling methods, reflecting the diversity of research designs and populations targeted. Some used prospective sampling; for example, Yoshifuji et al. (2020) used this method to ensure the inclusion of a representative sample of hematopoietic stem cell transplant (HSCT) survivors [37] Reyna‐Figueroa et al. [31], and Ladas et al. [35] used convenience sampling to meet practical recruitment challenges. Investigators used stratified sampling to capture specific subgroups within the study population; for example, Stein‐Thoeringer et al. [30] stratified samples in their multicenter study by geographic location and center. The results of large‐scale studies like Stein‐Thoeringer et al.'s that recruited across multiple centers had enhanced generalizability due to the diversity of their survivor populations [30]. Single‐site recruitment was common in smaller studies or pilot trials, such as those by Andersen et al. [39] and Simona Stein et al. [29]. Some studies, such as Skaarud et al. [42] used secondary analysis of existing data to examine relationships between dietary habits, probiotic use, and treatment outcomes.

The result section is organized into six subsections based on HPLPII dimensions and the association of each domain with gut microbiota.

### 4.1 Nutrition

The majority of the studies examined the impact of nutritional interventions on the gut microbiota of survivors with hematological cancer or individuals after HSCT. A number of these looked at the effects of enteral nutrition (EN) alone or in comparison to parenteral nutrition (PN), probiotics, prebiotics, and other dietary modifications.

Iyama et al.'s retrospective comparative study demonstrated that enteral supplementation with glutamine, fiber, and oligosaccharides (GFO) reduced gut bacterial translocation and improved short‐term survival of patients after HSCT by alleviating gut injury and enhancing gastrointestinal health [27]. D'Amico et al. compared the effects of EN and PN in a longitudinal analysis of pediatric HSCT survivors [28]. They reported that EN promoted a more diverse and healthier gut microbiota, including a higher abundance of SCFA‐producing bacteria. These effects were associated with fewer systemic infections and a reduced incidence of GvHD. However, Andersen et al. in a pilot study that was part of a larger RCT, found no significant difference in microbial diversity between adult HSCT survivors receiving EN versus PN. The discrepancy may be due to differences in patient age groups, study design, and sample size. EN was, however, associated with a greater abundance of beneficial taxa, suggesting potential long‐term benefits of EN in maintaining a healthy gut microbiota post‐transplant.

Another group of studies looked at the effects of consumption of probiotics and reported inconsistent results. Reyna‐Figueroa et al. conducted a randomized pilot study to assess the effects of probiotic supplementation in children with acute leukemia undergoing chemotherapy [31]. The results indicated that *Lactobacillus rhamnosus* GG effectively reduced the prevalence and severity of gastrointestinal side effects, suggesting that probiotics can help restore gut microbiota balance disrupted by chemotherapy.

However, Gorshein et al. in an RCT studying the impact of *Lactobacillus rhamnosus* GG supplementation on gut microbiota and GvHD incidence in survivors of allo‐HCT, found no significant differences in gut microbiota diversity or GvHD incidence between the probiotic and control groups. This inconsistency could stem from differences in probiotic strains, patient populations, or treatment durations [38]. In another pilot study, Ladas et al. evaluated the safety and feasibility of administering *Lactobacillus plantarum* (LPB) to children undergoing allo‐HCT [35]. While they found that probiotic administration was safe, with no cases of LBP bacteremia, the impact on overall gut microbiota composition was insignificant, possibly due to limited colonization or individual variations in microbiome responses.

The effect of consuming dietary fiber, or the subset of fibers designated as prebiotics, on gut microbiota was another area of study. D'Angelo et al.'s study among multiple myeloma survivors undergoing autologous stem cell transplantation revealed that increased intake of dietary fiber is associated with greater gut microbiota diversity and improved treatment responses [32]. In a prospective study, Shah et al. reported that greater consumption of dietary flavonoids, and prebiotics found in some plant‐based foods, was associated with higher concentrations of the SCFA butyrate in the stool, greater stool microbiota diversity, and sustained minimal residual disease (MRD) negativity in multiple myeloma survivors on lenalidomide maintenance [33].

In another prospective study among patients undergoing allo‐HSCT, Shah et al. found that prebiotic intake preserved microbial diversity and increased the population of butyrate‐producing bacteria, leading to reduced oral mucositis and diarrhea and a lower incidence of GvHD [37]. Later Shah et al. in another study treated patients with a high‐fiber diet, which resulted in increased alpha‐diversity and butyrate producers [34]. Similarly, Paredes et al. reported that patients with high fiber intake displayed higher microbial alpha diversity and butyrate producers [36]. Despite these positive findings, individual variations in microbiome composition, dietary adherence, and differences in fiber types consumed may influence outcomes across studies.

Riwes et al. found that a dietary intervention using resistant potato starch (RPS), a prebiotic, among allo‐HCT recipients significantly increased intestinal butyrate levels, favorably modulating the gut microbiome without adverse effects [41, 43]. This study provided preliminary evidence that RPS is a feasible intervention to improve gut health in allo‐HCT survivors [41, 43]. However, Andermann et al. in a Phase I trial, found that intake of the prebiotic fructooligosaccharides (FOS) was linked with transient changes in the gut microbiota and increased concentration of peripheral regulatory T cells, though no significant clinical differences were observed. The variations in outcomes might be due to differences in prebiotic type, study duration, or host metabolic responses [40].

Lastly, in their multicenter study that combined human cohort studies with experimental mouse models, Stein‐Thoeringer et al. reported that *Enterococcus* expansion in the gut microbiota was associated with increased GvHD risk and mortality post‐allo‐HCT [30]. Findings revealed that a lactose‐free diet could mitigate *Enterococcus* outgrowth and subsequent GvHD. This suggests that dietary interventions can influence gut microbiota composition, but further research is needed to clarify the mechanisms and long‐term effects.

### 4.2 Graft‐Versus‐Host Disease

In patients with Graft‐versus‐host disease (GvHD), a complication associated with allogeneic hematopoietic stem cell transplantation (allo‐HCT), presents a unique and complex context for examining gut microbiota alterations [24, 44]. The intestinal damage induced by preconditioning and conditioning chemotherapies can inflame GvHD and impair nutritional status, which exacerbates this condition [45]. Patients with GvHD often experience decreased microbial diversity and alterations in microbial composition, which are predictors of poor prognosis [24]. Studies have shown that a healthy nutritional status and a well‐maintained gut barrier are protective factors against GvHD, and poor nutritional status and reduced plasma citrulline levels, the most sensitive indicator of intestinal barrier health, predict the likelihood of developing acute GvHD following allogeneic hematopoietic cell transplantation (allo‐HCT) [45].

Nutritional interventions, particularly enteral nutrition (EN) and parenteral nutrition (PN) have been explored for their potential roles in modulating the gut microbiota in GvHD patients. EN has shown superior promise over PN in promoting the abundance of short‐chain fatty acid (SCFA)‐producing bacteria, which support gut barrier integrity and modulate immune responses [46]. This may help mitigate GvHD severity and improve clinical outcomes. However, it is essential to note that patients requiring PN often experience more severe GvHD or systemic disease [47, 48]. These underlying conditions, rather than PN itself, may primarily drive negative changes in the microbiome; for example, *Blautia* was associated with reduced GvHD mortality, and patients who received PN displayed the loss of *Blautia* in their gut microbiota. Thus, while PN is a critical supportive therapy for patients unable to tolerate oral or enteral feeding, its effects on gut microbiota should be interpreted cautiously in the context of disease severity.

The variability in microbiome responses to probiotics, dietary fiber, and prebiotics in GvHD patients underscores the complexity of this condition. Probiotic use in GvHD has yielded inconsistent results; it has been reported that probiotic consumption reduced the duration of oral mucositis and diarrhea, as well as the incidence and severity of aGVHD. Moreover, in patients who consumed prebiotics, microbial diversity, butyrate‐producing populations, and butyrate levels were preserved [37]. Additionally, probiotics were shown to increase regulatory T cells (Tregs), which play a vital role in immune regulation and prevent excessive immune responses that can lead to aGVHD [49]. However, some studies have reported no effect on the GVHD rate or infection, including treating patients with a mix of FOS and oligosaccharides or Glutamine and GFO [27, 49, 50]. This variability may reflect strain‐specific effects, patient heterogeneity, or interactions with other therapies, such as immunosuppressive drugs. Further research is necessary to identify optimal strains and dosages supporting gut health in GvHD patients.

These interventions may offer a supportive role in improving gut health and reducing GvHD complications. However, the individualized responses observed in studies highlight the need for personalized approaches and robust clinical trials to validate these findings in GvHD populations. Random sampling. In summary, GvHD represents a unique and complex condition with distinct microbiome and therapeutic considerations. Nutritional and microbiome‐targeted interventions, such as EN, probiotics, and prebiotics, hold promise but require careful interpretation within the broader context of disease severity and individual variability. Further research is essential to optimize these strategies and improve outcomes for patients with GvHD.

### 4.3 Physical Activity

Only one study looked at the effects of physical activity on the gut microbiota in survivors of hematological cancer. In their prospective cohort study investigating Simona Ugrayová et al. (2022) investigated the impact of HSCT on the gut microbiota in children with acute lymphoblastic leukemia (ALL), Ugrayová et al. found a significant decline in bacterial diversity post‐HSCT along with an increase in the prevalence of pathogenic bacteria like *Enterococcus* spp. Reduced microbial diversity and increased pathogenic bacteria are often associated with negative health outcomes, including infections and inflammatory conditions [29]. However, the study also uncovered an intriguing finding: physical activity was positively correlated with bacterial diversity. Children who engaged in regular physical exercise showed a more diverse gut microbiota compared to those who were less active, suggesting that exercise may help counteract some of the negative effects of HSCT on the gut microbiota. The study did not provide specific details on the types, intensity, or frequency of the physical activity sessions that were most beneficial. Further research is needed to determine the optimal exercise regimen, including the number of sessions per week and the types of physical activity (e.g., aerobics, strength training) that are most effective in promoting gut microbiota diversity in pediatric survivors post‐HSCT. Understanding these specifics could help in designing targeted interventions to maximize the health benefits of exercise for children undergoing HSCT.

### 4.4 Health Responsibility

Health responsibility encompasses an individual's commitment to maintaining and promoting their health through behaviors such as creating good health habits, self‐reflection, and aligning with societal health norms. This multidimensional concept highlights the importance of engagement in health‐promoting activities, self‐awareness, and treating health as a moral obligation [51]. However, further research is necessary to better understand the specific impacts of health responsibility on individual health outcomes and the roles of education and social factors in shaping these behaviors.

The relationship between health responsibility and gut health is an emerging area of interest. While no direct link between the two has been established in the current literature, behaviors associated with health responsibility, such as maintaining a balanced diet, regular exercise, stress management, and probiotic use are known to positively influence gut microbiota. A healthy gut microbiome is linked to benefits like enhanced immune function, improved digestion, and reduced risk of chronic diseases [52, 53, 54]. These observations suggest that health‐conscious individuals may indirectly promote gut health through their lifestyle choices, though more research is required to confirm this connection.

The gut microbiome's role in immune function, inflammation regulation, and metabolic processes is well‐documented [55]. Health‐responsible individuals may adopt behaviors supportive of gut health, but the extent and implications of this association remain unclear. Future studies should focus on directly linking health responsibility behaviors to gut health and investigating the mechanisms underlying these relationships to inform effective health interventions.

### 4.5 Spiritual Growth

Spiritual practices, such as meditation, may influence gut microbiota composition and diversity, potentially affecting the gut‐brain axis. Cyclic meditation (CM), for example, has been associated with stress reduction and improved vagal tone, factors that can modulate gut microbiome dynamics. Preliminary studies suggest a correlation between long‐term CM practice and the abundance of beneficial gut bacteria, such as *Roseburia, Subdoligranulum*, and *Lachnospiraceae* [56]. While these findings are promising, additional research is needed to confirm and elucidate these associations.

Mindfulness meditation and contemplative practices may also foster a balanced microbial environment, potentially promoting microbial diversity. Historical and cultural practices, such as religious rituals and Amerindian shamanic traditions, have emphasized a connection between spiritual well‐being and microbiome health, often incorporating antimicrobial herbs and cleansing rituals [17]. These traditions underscore the historical recognition of the microbiome's role in health, though scientific validation of such claims remains limited.

Emerging evidence suggests that mindfulness practices may benefit gut health by reducing inflammation and modulating gene expression along the gut‐brain axis, improving symptoms of conditions like irritable bowel syndrome (IBS) and inflammatory bowel disease (IBD) [57]. Additionally, certain spiritual practices, such as Spiritist “passe,” have been observed to affect microbial growth in laboratory settings, though the mechanisms are not well understood [22].

While these findings hint at the potential interplay between spirituality and gut health, further studies are essential to establish causation, explore underlying mechanisms, and determine the broader implications for health and disease management.

### 4.6 Interpersonal Relationships

Research has demonstrated a strong correlation between close social relationships and the composition of the gut microbiota. For instance, married individuals and those in close relationships exhibit greater diversity and richness in their gut microbiota compared to those in less close relationships or living alone. PEVuZE5vdGU [22] This relationship is bidirectional; the gut microbiota influences the host's stress response, while the host's stress levels impact the gut microbiota [58, 59]. Better relationship quality is associated with greater microbial diversity [60]. In addition, it has been shown that cohabiting romantic partners tend to have more similar gut microbiota compositions compared to unrelated individuals or siblings. PEVuZE5vdGU [60, 61] Moreover, it has been shown that our well‐being is deeply intertwined with the health of our microbial partners, who possess remarkable capabilities such as consciousness, cognition, and inter‐kingdom connectivity [17]. This similarity is likely due to factors such as the sharing of microbiota through physical contact (e.g., kissing, touching) [61]; shared living environment and exposure to common environmental microbes [60, 61]; and concordance in dietary habits and other health behaviors that shape gut microbiota [62]. However, the association of interpersonal relationships with the gut microbiota in hematological cancer is not yet defined, and further in‐depth investigation into these populations is needed.

### 4.7 Stress Management

Chronic stress, such as that experienced in dominant and subordinate relationships, can alter the gut microbiota [63]. Exposure to stressful stimuli can lead to gut microbiota dysbiosis through various pathways, including altered health behaviors, increased inflammation, and impaired gut barrier function [62], characterized by a reduction in *Lactobacillus* bacteria, and the gut microbiota can contribute to stressor‐induced immunomodulation [64]. On the other hand, it has been shown that mindfulness‐based stress reduction techniques have been associated with increased microbial diversity and changes in the relative abundance of certain bacterial taxa in the gut.

## 5 Discussion

Studies included in the current scoping review investigating associations between health‐promoting lifestyle behaviors and gut microbiota alterations in hematological cancer survivors used a range of research designs, including retrospective comparative studies, longitudinal studies, RCTs, pilot studies, prospective cohort studies, and observational studies, suggesting a comprehensive and multifaceted approach. The large majority of studies (15 of 16) investigating the association between health‐promoting lifestyle behaviors and gut microbiota alterations in hematological cancer survivors focused on nutrition. These studies collectively emphasized the critical role of nutrition in modulating the gut microbiota and improving health outcomes in hematological cancer survivors, including those who had undergone HSCT, allo‐HCT, and autologous stem cell transplantation. Nutritional interventions examined in these studies included EN, PN, probiotics, dietary fiber, prebiotics, and other dietary modifications. Findings across studies showed that EN was associated with a higher abundance of SCFA‐producing survivors, suggesting that prioritizing EN for HSCT survivors, particularly formulations with GFO, may improve gut health and reduce treatment complications. Continuous education and training on the benefits and administration of EN may thus enhance the care of survivors. The results regarding probiotics, on the other hand, were inconsistent, with some studies showing beneficial effects on gut microbiota balance and others indicating no significant impact. It may be that the protective effects of probiotics are strain‐specific or influenced by other factors. Further research on optimal probiotic strains and dosages, as well as other factors that alter the effects of probiotics is warranted. Studies also explored the effect of dietary fiber on gut microbiota in hematological cancer survivors. They found that dietary fiber intake was associated with the abundance of beneficial bacteria and gut microbiota diversity as well as improved treatment responses. Likewise, studies on prebiotics, such as RPS and FOS, showed promising results for preserving microbial diversity and increasing beneficial bacteria populations. Further research is needed, however, to determine optimal dosages and long‐term effects [40]. While EN, probiotics, high‐fiber diets, and prebiotics all showed promise, the variability in individual responses underscores the need for personalized approaches and further research to optimize these interventions for better clinical outcomes.

We found only one study that examined the role of physical activity in altering the gut microbiota in hematological cancer survivors. Investigators found that regular physical exercise correlated with increased gut microbiota diversity in children undergoing HSCT. However, the study did not specify the most beneficial intensity or frequency of physical activity. Further research is needed to determine the optimal exercise regimen, including the number of sessions per week and the types of physical activity (e.g., aerobics, strength training) that are most effective in promoting gut microbiota diversity in pediatric survivors post‐HSCT. This knowledge would support the design of targeted interventions to maximize the health benefits of exercise for children undergoing HSCT.

Considering analytical approaches, studies that employed the Wilcoxon Rank‐Sum Test consistently highlighted the role of dietary interventions in shaping gut microbiota composition and improving health outcomes post‐HSCT. D'Amico et al. demonstrated that enteral nutrition (EN) promoted microbial diversity and SCFA production, reducing infections in pediatric patients [28], while Shah et al. linked high‐fiber diets to sustained minimal residual disease (MRD) negativity in multiple myeloma through butyrate production [33]. In contrast, Gorshein et al. found that probiotics had minimal impact on microbial composition or GvHD prevention [38], and D'Angelo et al. noted that although pre‐transplant fiber increased diversity, post‐HSCT antibiotics negated these benefits, linking lower diversity to poorer outcomes [32]. Studies using the Kruskal‐Wallis Test, such as Iyama et al. and Stein‐Thoeringer et al. emphasized the influence of specific nutritional modifications EN with glutamine‐fiber‐oligosaccharides and lactose restriction, respectively, on reducing Enterococcus overgrowth and improving survival or mitigating GvHD risk [27, 30]. ANOVA‐based studies, including Ugrayová et al. reaffirmed the negative impact of HSCT on microbial diversity, with exercise emerging as a potential modulator [29]. Correlation‐based approaches, such as those by Ugrayová et al. and Shah et al. [29, 33], further supported the association between dietary and lifestyle interventions with enhanced microbial diversity and improved metabolic and inflammatory markers. Finally, Bray‐Curtis and UniFrac analyses, as applied by Stein‐Thoeringer et al. and D'Angelo et al. [30, 32], reinforced how dietary modifications (lactose restriction, fiber intake) could reshape gut microbiota composition pre‐ and post‐HSCT, though antibiotic use remained a disruptive factor. Collectively, these findings underscore the consistent benefits of targeted nutritional strategies and physical activity in fostering a resilient gut microbiota post‐transplant, whereas probiotic efficacy remains variable and antibiotic exposure may counteract microbiota‐driven benefits.

The review identified no studies directly addressing health responsibility, spiritual growth, interpersonal relationships, or stress management, though research suggests that these factors can influence gut microbiota diversity and composition. Health‐responsible individuals are more likely to engage in behaviors that support gut health, such as a balanced diet, regular exercise, and stress management. These behaviors can enhance gut barrier integrity, increase the production of beneficial microbial metabolites such as short‐chain fatty acids (SCFAs), and reduce systemic inflammation [59, 60, 61]. Spiritual practices, including meditation, may positively influence gut microbiota through the gut‐brain axis by modulating the autonomic nervous system, reducing cortisol levels, and promoting the growth of beneficial microbial species involved in neurotransmitter synthesis [17, 47]. Close social relationships have been correlated with greater gut microbiota diversity, likely due to shared environmental factors, diet, and microbiota transfer through physical interactions [24, 44, 65]. Oxytocin release during positive social interactions may also contribute to microbiome stability and immune modulation [24]. Conversely, chronic stress can negatively impact gut microbiota by altering microbial composition, increasing gut permeability, and triggering an overactive hypothalamic–pituitary–adrenal (HPA) axis, leading to dysbiosis [45]. While these factors have demonstrated potential in shaping gut microbiota, further research is needed to elucidate their specific mechanisms and relevance for hematological cancer survivors.

Overall, the reviewed studies underscore the critical role of nutritional interventions and physical activity in modulating gut microbiota and improving health outcomes in hematological cancer survivors. While health responsibility, close social relationships, spiritual practices, and stress‐management strategies have been associated with beneficial shifts in gut microbiota, the specific mechanisms of these relationships and their relevance to hematological cancer survivors remain to be fully elucidated.

## 6 Study Limitations

This review's focus on survivors of the full range of hematological cancers may limit the specificity of the findings to particular cancer types. Additionally, our review included only English‐language studies, potentially excluding relevant research published in other languages. Some of the included studies had limitations such as small sample size, lack of control, short follow‐up period, and inconsistent definition of the measure of gut microbiota changes that could impact the validity of their results. Consequently, the results may be subject to reporting bias. Differences between studies in treatment regimens and time points along the survivorship trajectory add variability to the findings. One dependent reviewer primarily screened abstracts; however, the iterative process of refining the inclusion and exclusion criteria helped achieve a more targeted review by reducing potential ambiguity, considering the broad scope of the research question.

## 7 Conclusion

This review examined the literature on the association between health‐promoting lifestyle behaviors and gut microbiota alterations in hematological cancer survivors. Nutritional interventions, particularly EN with targeted supplements, showed promise in improving gut health and reducing complications of treatment. The impact of probiotics was inconsistent across studies, suggesting the need for further research on strain‐specific effects. Dietary fiber and prebiotics demonstrated beneficial effects on gut microbiota diversity, though optimal dosages and long‐term outcomes require further investigation. The variability in individual responses to these interventions highlights the need for personalized approaches and further research to optimize strategies for better clinical outcomes. Physical activity was associated with increased gut microbiota diversity, but specific recommendations for exercise regimens remain to be determined. The lack of studies on the relationships between health responsibility, stress management, interpersonal relationships, and spiritual growth to alterations in the gut microbiota in survivors of hematological cancers indicates the need for comprehensive research in these areas. In synthesizing this body of research, this review is meant to support healthcare providers, researchers, and clinicians in developing tailored holistic interventions to enhance the care and management of hematological cancer patients and improve the health and well‐being of survivors.

## Author Contributions

**Elham Samami:** lead author; conceptualized the review, developed methodology, conducted the literature search, performed data synthesis, and drafted the manuscript. **Angela Starkweather:** provided critical feedback on methodology, data interpretation, and manuscript clarity. **Debra Lynch Kelly:** reviewed and provided feedback on data interpretation, implications, and manuscript revisions. **Debra Lyon:** supervised the edition and review process and provided feedback on manuscript structure and quality.

## Conflicts of Interest

The authors declare no conflicts of interest.

## Supporting information


**Table S1.** Comprehensive Search Strategy.

## Data Availability

Data sharing not applicable to this article as no datasets were generated or analysed during the current study.
