# Supplementary material for: The Impact of Health‐Promoting Lifestyle Behaviors on Gut Microbiota in Survivors of Hematological Cancer: A Scoping Review
Source: Cancer Rep (Hoboken). 2025 May 13;8(5):e70224. doi: 10.1002/cnr2.70224 (PMC12075932; doi:10.1002/cnr2.70224)
Supplement: Supplementary file 1 — Table S1. Comprehensive Search Strategy. [file CNR2-8-e70224-s001.docx]

**Supplemental Material**

**Table S1.** Comprehensive Search Strategy

| **PubMed** |
| --- |
| ("probiotics" [MeSH Terms] OR "prebiotics" [MeSH Terms] OR "microbiota" [MeSH Terms] OR "gut microbiota" [tiab] OR "gastrointestinal microbiome" [tiab] OR "microflora" [tiab] OR "microbiome" [tiab] OR Intestinal Microbiota OR Intestinal Flora) AND ("hematologic malignanc*" [Title/Abstract] OR "hematological cancer" [Title/Abstract]  OR "hematologic neoplasm" [Title/Abstract] OR leukemia* [Title/Abstract] OR lymphoma* [Title/Abstract] OR "multiple myeloma" [Title/Abstract] OR "myelodysplastic" [Title/Abstract] OR "myeloproliferative" [Title/Abstract] OR myelo*[Title/Abstract] OR Hematopoietic Stem Cell Transplant* OR HSC therapy OR HSC transplantation OR hematopoietic cell transplant* OR HSCT OR HEMATOPOIETIC STEM CELLS OR Allogeneic OR Allograft OR Homologous OR Autologous stem cell transplant* OR Autologous stem cell therap* OR Autologous hematopoietic stem cell transplant* OR Autologous peripheral blood stem cell transplant* OR Bone Marrow OR Graft* OR Peripheral Blood Stem Cell Transplant* OR Allogeneic Peripheral Blood Stem Cell Transplant* OR Autologous Peripheral Blood Stem Cell Transplant* OR Bone Marrow Transplantation OR Bone Marrow OR Peripheral Blood Stem Cell Transplantation)AND (Physical Activity [Title/Abstract] OR activity [Title/Abstract] OR Exercise [Title/Abstract] OR physical exercise [Title/Abstract] OR sport [Title/Abstract] OR fitness [Title/Abstract] OR walk* [Title/Abstract] OR aerobic activity [Title/Abstract] OR aerobic fitness [Title/Abstract] OR "cardiovascular exercise" [Title/Abstract] OR "cardiovascular fitness" [Title/Abstract] OR "cardiorespiratory exercise" [Title/Abstract] OR "cardiorespiratory fitness" [Title/Abstract] OR "physical education" [Title/Abstract] OR "sports program" [Title/Abstract] OR "curcumin" [tiab] OR "boswellic acid" [tiab] OR "arginin*" [tiab] OR "tendisulfur" [tiab] OR "bromelain" [tiab] OR "methylsulfonylmethane" [tiab] OR "amino acid" [tiab] OR "protein" [tiab] OR "leucine" [tiab] OR "glutamine" [tiab] OR "arginine" [tiab] OR "taurine" [tiab] OR "gelatin" [tiab] OR "collagen" [tiab] OR "phytochemicals" [tiab] OR "phytonutrients" [tiab] OR "coenzyme Q10" [tiab] OR "omega-3" [tiab] OR "lipids" [tiab] OR "fatty acids" [tiab] OR "fish oils" [tiab] OR "plant oils" [tiab] OR "diet therapy" [tiab] OR "nutrient intake" [tiab] OR "nutrition" [tiab] OR "micronutrient" [tiab] OR "vitamin c" [tiab] OR "antioxidant" [tiab] OR "vitamin d" [tiab] OR "cholecalciferol" [tiab] OR "ergocalciferols" [tiab] OR "minerals" [tiab] OR "calcium" [tiab] OR "manganese" [tiab] OR "copper" [tiab] OR "zinc" [tiab] OR "magnesium" [tiab] OR "iron" [tiab] OR "molybdenum" [tiab] OR "silicon" [tiab] OR "calories" [tiab] OR "dietary supplements" [tiab] OR "food supplements" [tiab] OR "nutraceutical" [tiab] OR "glycerin" [tiab] OR "glycerol" [tiab] OR "spiritual well-being" [Title/Abstract] OR "spiritual health" [Title/Abstract] OR Stress [Title/Abstract] OR "Stress Management" [Title/Abstract] OR well-being [Title/Abstract] OR stress* [Title/Abstract] OR "Stress " [MeSH Terms] OR "Anxiety" [MeSH Terms] OR anxiety [tiab] OR worry [tiab] OR Manage* [tiab] OR coping [tiab] OR cope* [tiab] OR "stress management" [tiab] OR technique* [tiab]Health [tiab] OR behaviour [tiab] OR knowledge [tiab] OR attitude [tiab] OR perception [tiab] OR consumption [tiab]) NOT ("review" [Title/Abstract]) NOT (rat [Title/Abstract]) NOT (mice [Title/Abstract]) NOT (cell [Title/Abstract]) |
| **Scopus** |
| (TITLE-ABS-KEY("Physical Activity" OR "activity" OR "Exercise" OR "physical exercise" OR "sport" OR "fitness" OR "walk*" OR "aerobic activity" OR "aerobic fitness" OR "cardiovascular exercise" OR "cardiovascular fitness" OR "cardiorespiratory exercise" OR "cardiorespiratory fitness" OR "physical education" OR "sports program") AND TITLE-ABS-KEY("probiotics" OR "prebiotics" OR "microbiota" OR "gut microbiota" OR "gastrointestinal microbiome" OR "microflora" OR "microbiome" OR "Intestinal Microbiota" OR "Intestinal Flora") AND TITLE-ABS-KEY("leukemia" OR "lymphoma" OR "multiple myeloma" OR "myelodysplastic" OR "myeloproliferative" OR "myelo*") AND TITLE-ABS-KEY("curcumin" OR "boswellic acid" OR "arginin*" OR "tendisulfur" OR "bromelain" OR "methylsulfonylmethane" OR "amino acid" OR "protein" OR "leucine" OR "glutamine" OR "arginine" OR "taurine" OR "gelatin" OR "collagen" OR "phytochemicals" OR "phytonutrients" OR "coenzyme Q10" OR "omega-3" OR "lipids" OR "fatty acids" OR "fish oils" OR "plant oils" OR "diet therapy" OR "nutrient intake" OR "nutrition" OR "micronutrient" OR "vitamin c" OR "antioxidant" OR "vitamin d" OR "cholecalciferol" OR "ergocalciferols" OR "minerals" OR "calcium" OR "manganese" OR "copper" OR "zinc" OR "magnesium" OR "iron" OR "molybdenum" OR "silicon" OR "calories" OR "dietary supplements" OR "food supplements" OR "nutraceutical" OR "glycerin" OR "glycerol" OR "spiritual well-being" OR "spiritual health" OR "stress" OR "Stress Management" OR "well-being" OR "stress*" OR "Anxiety" OR "anxiety" OR "worry" OR "Manage*" OR "coping" OR "cope*" OR "stress management" OR "technique*" OR "Health" OR "behaviour" OR "knowledge" OR "attitude" OR "perception" OR "consumption") AND NOT TITLE-ABS-KEY("review" OR "rat" OR "mice" OR "cell")) |
| **CINAHL** |
| (probiotics OR prebiotics OR microbiota OR gut microbiota OR gastrointestinal microbiome OR microflora OR microbiome OR Intestinal Microbiota OR Intestinal Flora) AND (hematologic malignan* OR hematological cancer OR hematologic neoplasm OR leukemia* OR lymphoma* OR multiple myeloma OR myelodysplastic OR myeloproliferative OR myelo* OR Hematopoietic Stem Cell Transplant* OR HSC therapy OR HSC transplantation OR hematopoietic cell transplant* OR HSCT OR HEMATOPOIETIC STEM CELLS OR Allogeneic OR Allograft OR Homologous OR Autologous stem cell transplant* OR Autologous stem cell therap* OR Autologous hematopoietic stem cell transplant* OR Autologous peripheral blood stem cell transplant* OR Bone Marrow OR Graft* OR Peripheral Blood Stem Cell Transplant* OR Allogeneic Peripheral Blood Stem Cell Transplant* OR Autologous Peripheral Blood Stem Cell Transplant* OR Bone Marrow Transplantation OR Bone Marrow OR Peripheral Blood Stem Cell Transplantation) AND (Physical Activity OR activity OR Exercise OR physical exercise OR sport OR fitness OR walk* OR aerobic activity OR aerobic fitness OR cardiovascular exercise OR cardiovascular fitness OR cardiorespiratory exercise OR cardiorespiratory fitness OR physical education OR sports program OR curcumin OR boswellic acid OR arginin* OR tendisulfur OR bromelain OR methylsulfonylmethane OR amino acid OR protein OR leucine OR glutamine OR arginine OR taurine OR gelatin OR collagen OR phytochemicals OR phytonutrients OR coenzyme Q10 OR omega-3 OR lipids OR fatty acids OR fish oils OR plant oils OR diet therapy OR nutrient intake OR nutrition OR micronutrient OR vitamin c OR antioxidant OR vitamin d OR cholecalciferol OR ergocalciferols OR minerals OR calcium OR manganese OR copper OR zinc OR magnesium OR iron OR molybdenum OR silicon OR calories OR dietary supplements OR food supplements OR nutraceutical OR glycerin OR glycerol OR spiritual well-being OR spiritual health OR Stress OR Stress Management OR well-being OR stress* OR Stress OR Anxiety OR anxiety OR worry OR Manage* OR coping OR cope* OR stress management OR technique* OR Health OR behaviour OR knowledge OR attitude OR perception OR consumption) NOT (review) NOT (rat) NOT (mice) |
